# Learning to attenuate myself: a predictive processing account of body-scan meditation and the dissolution of bodily boundaries

**DOI:** 10.1093/nc/niag001

**Published:** 2026-02-19

**Authors:** Valeria Becattini, Michael Lifshitz, Mark Miller

**Affiliations:** Linguistics & Philosophy IUSS Center (L&PIC), Scuola Universitaria Superiore IUSS, Palazzo del Broletto Piazza della Vittoria 15, 27100 Pavia (PV), Italy; Institute for Advanced Consciousness Studies, 2811 Wilshire Blvd, Santa Monica, CA 90403, United States; Division of Social and Transcultural Psychiatry, McGill University, 1033 Ave des Pins Ouest, Montreal, QC H3A 1A1, Canada; Lady Davis Institute for Medical Research, Jewish General Hospital, Montreal, QC H3T 1E1, Canada; Monash Centre for Consciousness and Contemplative Studies, Monash University, Melbourne, 29 Ancora Imparo Wy, Clayton VIC 3168, Australia

**Keywords:** meditation, predictive processing, attention, perception, interoception, self-regulation, well-being

## Abstract

Meditation practices often involve sustaining attention on the body. Typically, attention is understood to enhance both neural resource allocation and the subjective salience of the attended target. However, in deep meditative states, practitioners sometimes report a dissolution of bodily boundaries, a phenomenon known in Pali as bha${\dot{\textrm{n}}}$ga. This presents a paradox: why does focused attention, which typically heightens sensory perception, instead lead to its dissolution? This article addresses this apparent contradiction by integrating computational, phenomenological, and empirical perspectives on attention, interoception, and meditation. We focus on the body-scan technique, as practiced in Theravada Buddhist traditions, and its powerful capacity to produce experiences of the dissolution of bodily boundaries. Working within the predictive processing framework, we propose that this “dissolution” of bodily boundaries results from the body-scan’s impact on attentional processes. We argue that by optimizing low-level predictions over somatosensory signals, the body-scan effectively attenuates these signals, thereby diminishing perception of the body’s boundaries. In support of this claim, we first describe the body-scan technique and its phenomenological outcomes. We then introduce key concepts from the predictive processing framework and provide a detailed analysis of attentional processes during the body-scan. We conclude that the attenuation of somatosensory signals during the body-scan not only contributes to the experience of bha${\dot{\textrm{n}}}$ga but also suggests a broader potential of this practice for enhancing well-being. With appropriate therapeutic integration, this attentional modulation offers promising applications in addressing conditions characterized by disrupted self-regulation, such as addiction and emotional dysregulation.

## Introduction

The practice of mindfulness of the body holds a primary role in the Theravāda Buddhist tradition *via* the influential texts of the Satipaṭṭhāna-sutta (Anālayo 2003). The Buddhist emphasis on the body in contemplative practice has led to a variety of meditation techniques, including the body-scan.

The body-scan is a meditation technique consisting of systematically engaging attention throughout the body. As listed in one of the Pali instructional manuals of the Theravāda tradition, the Abhidhammattha-sa${\dot{\textrm{n}}}$gaha, the body-scan leads to 10 different stages of insight (for a comprehensive scheme of the insight dynamics, see Anālayo 2012). Among them, the stage of “dissolution” (in Pali, bha${\dot{\textrm{n}}}$ga) has gained attention in the literature of cognitive science, also known as “body boundaries dissolution” (Berkovich-Ohana et al. 2013; Hanley et al. 2020; Nave et al. 2021; Ciaunica 2024). Empirical studies seem to confirm that the body-scan tends to lead to a decrease in the perception of body boundaries (Dambrun 2016; Dambrun et al. 2019).

Through constant practice, the meditator is said to begin to understand the impermanence of existence (stage 1) and to develop “knowledge of rise and fall” (stage 2) (Anālayo 2012:33). This eventually culminates in a dissolution of all phenomena pertaining to one’s subjective experience (stage 3). In other words, the meditator experiences all bodily sensations, thoughts, and emotions as gradually dissolving. This stage is followed by a series of realizations about one’s self (i.e. seen as unstable, dissatisfying, or “disenchanted”) and emotional life (i.e. development of a sense of equanimity toward extremes of positive and negative emotion).

In this article, we aim to investigate the body boundaries dissolution as led by the body-scan through the lens of cognitive science models of perception and action in the predictive processing (PP) framework (Friston 2005; Clark 2013, 2015; Hohwy 2013, 2016). This computational mechanistic perspective considers organisms to be prediction machines, endowed with causal models of the world that are continuously updated depending on variations in the environment (Hohwy 2013; Clark 2015). Previous accounts in PP have provided mechanistic models of the attentional process involved in meditative practices (Lutz et al. 2019; Pagnoni 2019; Deane et al. 2020; Limanowski and Friston 2020; Laukkonen and Slagter 2021; Pagnoni and Guareschi 2021; Ramstead et al. 2021; Laukkonen et al. 2023; Ciaunica 2024; Tal et al. 2025). Here we extend the PP framework to provide a computational account of the body-scan.

We are particularly interested in explaining how the consistent sweeping of attention through the body leads to the experience of body boundary dissolution. In most instances, allocating attention to a target tends to increase both its cortical and phenomenological response (Kok et al. 2012). Why then does sweeping attention throughout the body lead to a decreased perception of the body? By linking the phenomenology of the body-scan to a computational explanation of the underlying attentional processes, this article seeks to offer a unified mechanistic account of the body-scan practice.

Our main proposal is that practicing the body-scan facilitates and enhances the sensory adaptation of bodily sensations. In brief, we suggest that the body-scan enhances the accuracy of (low-level) predictions on somatosensory cues. Consequently, the resulting accurate posteriors accurately match the stream of ascendant prediction errors. Predictions matching prediction errors lead to what is called “sensory adaptation,” i.e. the attenuation of prediction errors over (somato) sensory signals due to highly expected signal (i.e. see, in detail, repetition suppression, Auksztulewicz and Friston 2016; Grotheer and Kovács 2016). The resulting overall reduced perception of the body of the practitioner may, in this way, render the phenomenology of the dissolution of body boundaries.

A second relevant question addressed in this article is how the processes underlying the dissolution of body boundaries relate to subjective well-being. Studies have shown that the body-scan can improve one’s life satisfaction (Kropp and Sedlmeier 2019), reduce biological markers of chronic stress (Schultchen et al. 2019), rumination, and anxiety, and increase levels of good mood (Dambrun 2016; Dambrun et al. 2019). We propose that the sort of attenuation to one’s bodily sensations promoted by the body-scan enables the opacification (or modeling) of the embodied self through interoceptive processing. We then highlight how the deep interoceptive modeling promoted by the body-scan technique may hold potential for optimizing therapeutic interventions, especially in the treatment of emotional dysregulation and addiction.

This article is structured as follows. First, we give a detailed description of the body-scan and its phenomenology, and introduce relevant concepts in PP, such as action, attention, perception, and self-modeling. Then, we propose a PP account of the attentional processes at work in the body-scan, and bridge our model to the phenomenology of body boundary dissolution. Finally, we highlight the relevance of this technique for the sense of self and the regulation of emotions and addictions.

## The body-scan meditation technique and its phenomenology

### Focused attention and open monitoring in the body-scan technique

The main aim of the body-scan technique is to maintain attention to all the sensations that arise inside the body (visceral, interoceptive) and outside the body (the contact with the clothes, surfaces, the air, etc.) (Hart 2011). Typically, with closed eyes and in a seated still position, the sustaining of attention is a continuous redirection of the focus away from distractions and back toward the bodily sensations, maintained with a non-judgmental and non-reactive attitude. The body-scan encompasses two common styles of meditation: focused-attention (FA) meditation, i.e. focus on a designated meditation object, and open monitoring (OM) meditation, i.e. broad non-judgmental focus on one’s field of experience (for a review, see Lutz et al. 2007, 2008; Lippelt et al. 2014).

The first phase of the body-scan consists of focusing attention on singular body parts, making sure to attentively “scan” in a systematic and repeated way. A commonly proposed way of doing this is to move the attention from the head to the feet and from the feet to the head. Once one is familiar with attending to single parts of the body, they may proceed to attending to multiple parts of the body in a symmetric and systematic fashion (e.g. both shoulders at the same time). During this phase, meditators typically meet some “blind spots”—areas where no sensations are felt (Khin 1999). Common examples are the central torso and the legs, which are not usually attended to in everyday life. Thus, this practice is ideal for increasing one’s interoceptive access to parts of the body that are commonly left unattended. This first phase continues as long as these blind spots are hardly perceived or not perceived at all.

We describe this first phase as a FA practice (Lippelt et al. 2014). FA meditation involves maintaining moment-to-moment selective attention on a specific object (e.g. the breath, physical sensations, etc.). Initially, attention may wander away from the chosen object, but the meditator is instructed to recognize the distraction and to bring their attention back to the meditation object. Crucial to achieving an ongoing focus on the intended object, the meditator must continually monitor the quality of attention.

In the second phase, the meditator attends to their body as a whole. As the practice progresses, the meditator begins to apply attention not only to individual parts but rather to the ongoing flow of sensations throughout the whole body. Now the meditator is able to perceive their body rapidly (without the need to attend to each part of the body separately) and easily (without any blind spots) (Anālayo 2003; Dambrun 2016). This phase is sometimes called the “free flow” (Khin 1999). Although the meditator is told about this second phase, they are typically advised not to imaginatively contrive their free flow or force themselves to feel the body as a whole. This phase is named as such because the meditator is able to flow freely with their attention throughout the body. They are instructed to come back to the first phase whenever they feel blind spots or lose their free flow, so that the practice often consists of an oscillation between FA and OM. This second phase is when the meditator is expected to feel their body boundaries dissolve, as they enter the state of bha${\dot{\textrm{n}}}$ga:

When the entire solidity of the body and of the mind is dissolved, then it is possible to move one’s attention from the head to the feet without any obstacle. In Pāli, this stage is called bha${\dot{\textrm{n}}}$ga, meaning dissolution, because there is no obstacle in any part. (Khin 1999:105)

We suggest that during the free flow phase of the body-scan, the practitioner senses their body as a whole using an OM style of attention. OM practices involve remaining attentive to anything that occurs in experience without focusing on any explicit object (Lutz et al. 2008; for a review, see Vago and Silbersweig 2012). Contrary to FA that prescribes a strong distinction between selection of the primary focus (e.g. the breath) and deselection of what should remain in the background (e.g. the emotional tone), typical OM practices allow monitoring different experiences without a specific one being the primary focus.

However, it is worth mentioning that in what we suggest to be the OM phase of the body-scan, the practitioner monitors their experiences with special emphasis on bodily sensations. This is not to say that OM practices do not target the body, but we can imagine that in the OM phase of the body-scan, the body represents the scope of awareness of the experiences. This means that OM is applied to a targeted scope of experience, i.e. the set of sensations occurring throughout the body, which are eventually sensed together at the same time.

Another important feature of the OM style of practice is that by broadening awareness of their ongoing experience, the practitioner diffuses their attention such that their salience distribution becomes increasingly flattened, so that ordinarily salient features of experience become less “provocative.” In other words, OM can lead the practitioner to what in Buddhist terms is referred to as equanimity. Equanimity (in Pali, upekkhā) involves one’s ability to regulate arousal—without falling into hyperexcitability or fatigue—by detaching from one’s responses of craving or aversion to ongoing sensations (Wallace 2006; see also Desbordes et al. 2015). That does not mean that the OM meditator does not experience feelings at all. Rather, OM promotes a way of leveling one’s salience landscape (the territory of relevant stimuli), which eventually enhances equanimity toward experiential patterns of attraction or aversion. As practitioners strive to evenly monitor the ongoing flux of experience, “awareness of the background of experience further comes to the foreground” (Laukkonen and Slagter 2021:204). In this way, sensations typically connected with craving or aversion become less salient or noteworthy because they are contextualized within a broader field of ever-changing, unstable sensations (see also Lutz et al. 2015).

### Changing the sense of body boundaries

Phenomenologists commonly distinguish between the physical body, characterized by anatomical boundaries, and the subjective body, characterized by the lived, first-person experience (Merleau-Ponty 1962; Husserl 1989). Both the physical body and the subjective body are subject to changes. For instance, when dreaming, the physical body undergoes sleep-related physiological changes (Vyazovskiy 2015), while the lived body changes according to one’s dream, e.g. by turning into the body of another animal (Solomonova 2018). Another example can be emotional experience, affecting physiological changes in the physical body (e.g. anger increasing one’s heartbeat), and in the subjective body (e.g. anger increasing salience in one’s face) (Craig 2002; Damasio and Carvalho 2013; Nummenmaa et al. 2014; Critchley and Garfinkel 2017).

In line with this distinction, we can think of one’s sense of body boundaries as a flexible experience that depends on different conditions and variables, from the environment to different senses (Ataria et al. 2015) and cognitive-emotional states. Pain can be a case of a specific sensation altering the perception of one’s body and its boundaries, as observed in cases of patients of chronic pain showing altered proprioception and sensitivity to external stimuli (for a review, see Tsay et al. 2015; for a discussion, see de Vignemont 2016). Moreover, different kinds of touch can convey different experiences of body boundaries. Grasping single objects is different from touching a “whole” environment (Ratcliffe 2013). In a qualitative study of 27 expert meditators, Yochai Ataria provides the following metaphor for the shift in their perception of bodily boundaries: “If I let myself gently sink into the water, with my eyes closed, the boundaries will become even less clear and more flexible. If, however, we open my eyes while sinking, the sense of boundaries will immediately strengthen once again” (Ataria 2015:1140). In this scenario, the person’s body is immersed in water, and this tactile experience conveys a sense of connectedness with the surroundings. Their body boundaries eventually shrink down once they change a condition of experience, for example, by opening their eyes.

Changes in the perception of body boundaries are commonly reported among meditators practicing body-scan techniques (Dambrun 2016). The most frequently reported phenomenon is decreased perception of body boundaries, often diminishing in varying degrees, eventually to the extent of being “almost imperceptible” (Dambrun 2016:93; Lindahl et al. 2017). These experiences, sometimes associated with terms such as “ego expansion” (Fink 2020), “oceanic boundlessness” (Dittrich 1998), or “dissolution of body boundaries” (Berkovich-Ohana et al. 2013), involve feeling blurred boundaries between the body and the external environment, or limbs “extended […] to the reaches of the universe” (Thompson 1994).

In addition to changes in the perception of body boundaries, meditators report a wide range of altered experiences, especially related to the sense of self (see Millière and Metzinger 2020). The sense of self, involving minimal (embodied) and narrative (reflective) components (Damasio 1999; Gallagher 2000; Zahavi 2005; James 2007; Zahavi and Kriegel 2015), dynamically changes during deep meditation—shrinking, expanding, dissolving, or merging with the environment (Berkovich-Ohana et al. 2013). Changes in the perception of the boundaries of one’s body may play an important role in facilitating these other, more general changes in self-concept and self-experience sought after by Buddhist contemplative programs, leading meditators to view self-related processes as more impermanent, flexible, and dynamic (Millière 2020; Millière and Metzinger 2020; Gallagher et al. 2024).

To sum up, in this section, we have described the body-scan as a systematic engagement of attention through the body that eventually leads to a decreased perception of body boundaries. We are now positioned to pose the following riddle: Why does increasing attention to bodily sensation eventually decrease the perception of the body’s boundaries? To answer this question, we will turn to the mechanistic framework of PP.

## The predictive processing framework

### Key concepts in predictive processing

The PP framework bridges cognitive phenomena (e.g. attention) with mechanistic accounts by explaining brain processes as hierarchical Bayesian models that update based on prediction errors (see, for example, Friston et al. 2011). Biological agents generate internal models (or “generative models”) to predict sensory inputs and then adjust these models when incoming signals conflict with the internal models (“prediction errors”; Clark 2013). Lower-level processes track these errors and inform higher levels to minimize them, creating a hierarchical, recurrent, and inferential loop. This model optimization maximizes the mutual information between sensory data and prior predictions, continually refining the system’s accuracy in interpreting the world (Friston 2010).

The system’s model optimization occurs on multiple timescales driven by prediction error states (knowledge-related update) and outcomes (action-related update) (Kiebel et al., 2008; Pezzulo et al. 2018; Smith et al. 2022). On slower timescales, errors adjust the generative model’s parameters, guiding abstract beliefs and long-term goals (Limanowski and Friston 2020). On faster timescales, errors enable moment-to-moment adjustments, such as sensory feedback and motor control. This equips organisms with a temporally deep generative model, where immediate outcomes inform higher-level inferences and long-term goals (Pezzulo et al. 2018).

Recently, ideas from the PP framework have developed into a distinct account explaining decision-making and actions as the result of inferential processes, also known as active inference theory (Friston 2009; Friston 2010; Brown et al. 2013; Sprevak and Smith 2023). Agents engaging in active inference update and fulfill expected priors not only about their current state but also about future states (Friston 2010). This means that agents do not only update their priors to better fit the sensory data but they can also “update” their sensory signals through action to better fit their priors. That is, agents can actively manipulate their environment (i.e. they update the world by acting on it) in order to maintain certain variables essential for their survival. These essential variables function as goal priors, like maintaining sugar concentration in the blood or a steady healthy body temperature. When prediction errors arise because of a discrepancy between an actual system state (i.e. low level of sugar) and the expected goal prior (i.e. sufficient level of sugar), the agent can fulfill this discrepancy by directly acting on the sensory data (i.e. eating a strawberry).

Following Bayesian schemes, prior beliefs and sensory data are probability distributions with a mean value (expectation) and precision or prediction of predictability (inverse variance) (Friston 2009). That is, precision is inversely proportional to the noise present in the signal, so that the precision of a variable is low if the average divergence from the variable’s mean is high. In terms of neural dynamics, precision reflects the postsynaptic gain of neurons that encode sensory data, and more abstract inferences and predictions at higher hierarchical levels. So, a high precision of the prediction error indicates that the sensory information is estimated to be reliable, while lower precision implies that prior predictions are considered more trustworthy.

If prediction errors are weighted according to sensory data and rely on weak priors, the mean of posteriors (updated beliefs) will be closer to the sensory data. Conversely, if the precision estimates are weighted according to strong priors and sensory data are relatively imprecise, posteriors will be closer to prior beliefs. Hence, estimates of precision are used to assign weights to prediction errors and thus determine how much these errors will impact the model updating at other layers in the predictive hierarchy. This precision weighting allows the inferential model to optimize how much it updates itself depending on the reliability of the incoming sensory data (Friston 2010; Adams et al. 2013).

In sum, agents adapt to and change the environment by trying to predict how likely or predictable events are to occur. To do so, they minimize the difference between the incoming sensory input, i.e. lower levels, and the priors, i.e. higher levels, based on their generative models. Agents anticipate potentially surprising states before they occur as well, by acting in order to minimize uncertainty about future outcomes, the core concept of the active inference theory (Friston et al. 2011). In what follows, we will refer to the PP as including its extension to active inference theory.

### Attention as precision weighting

Depending on the salience of sensory data, the precision assigned to prediction errors can increase or decrease. Optimizing precision estimates is equated to allocating attention (Friston and Stephan 2007; Feldman and Friston 2010; Hohwy 2020).

In cognitive science, attention is seen as a set of mechanisms for selecting and allocating information-processing resources (Knudsen 2007; Posner 2011, for a discussion on attention and perception, see Anderson 2011; Hommel et al. 2019; Wu 2024). Exogenous attention is bottom-up, involuntary, and stimuli-driven, while endogenous attention is top-down and voluntary (Egeth and Yantis 1997). In the framework of hierarchical inference, attention influences prediction errors by modulating the precision weighting of sensory data (Feldman and Friston 2010; Hohwy 2020).

Endogenous attention generates precision for targeted stimuli (Hohwy 2013; Hohwy 2020:12), as it selectively enhances the precision of sensory processing (Kok et al. 2012), aligning with known attention mechanisms (Treue and Trujillo 1999; Reynolds and Heeger 2009; Kok et al. 2012). In neural terms, top-down attention boosts post-synaptic responsiveness or contrast gain across cortical stages (Lou 1999; Posner and Gilbert 1999; Gazzaley and Nobre 2012).

In PP terms, introspective attention (attention toward one’s subjective experience) (Metzinger 2017; Limanowski and Friston 2018; Lutz et al. 2019) and covert attention (a change in attention that does not include a physical movement) (Sandved-Smith et al. 2021) have been defined as “mental actions.” These kinds of attentional processes are considered to be specific instances of the general mechanism of estimating precision in action control (Limanowski and Friston 2018:3), here applied to higher-level cognitive states. This is also described as a mechanism of policy selection over higher level cognitive states, i.e. weighting a set of mental actions chosen to minimize prediction error outcomes (i.e. prediction errors driving policy selection, for more details, see Smith et al. 2022), in a way comparable to motor control (Pezzulo et al. 2018; Sandved-Smith et al. 2021:1). Meditative practices have been defined as a type of mental action as they engage introspective and covert attention (Lutz et al. 2019; Deane et al. 2020; Pagnoni and Guareschi 2021). For instance, focusing attention on a bodily sensation, such as one’s breath, involves the mental action of estimating precision over interoceptive predictions to minimize prediction errors (Limanowski and Friston 2018).

### Predictive processing, self-modeling, and repetition suppression

Importantly, to construct an accurate model of the world, the generative model needs to differentiate between signals originating from agents themselves and those originating from the external world (Clark 2013). Crucial for apt self-modeling is the ongoing interoceptive feedback that the system receives as it continuously adapts to its changing niche (Metzinger 2004; Seth 2013; Tsakiris and Critchley 2016). In this context, it has been proposed that building self-representations largely depends on the process of precision weighting on proprioceptive, interoceptive, and exteroceptive signals (Seth et al. 2012; Seth and Critchley 2013). Think, for instance, of sitting in a café with a friend. Generally, the body or visceral sensations are not constantly attended to, but rather the perception of the friend has priority (e.g. related visual and auditory data gain more weight). However, internal signals can easily gain priority when needed, e.g. a feeling of thirst. Hence, ongoing adjustments to precision weighting help the brain and central nervous system (CNS) balance the extent to which external and internal somatosensory information determine the boundary between the self and the external world.

Central to this creation of a functional and phenomenological boundary is the perceptual attenuation of self-generated sensory signals. Formally, self-generated signals produce expectancy effects by successfully predicting related sensory consequences, thereby reducing the salience of sensations (Auksztulewicz and Friston 2016). This sensory adaptation is also called “repetition suppression” (Garrido et al. 2009; Auksztulewicz and Friston 2016; Grotheer and Kovács 2016). More precisely, repeated exposure to an identical stimulus elicits attenuated neural responses within the population encoding its representation, reflecting synaptic adaptation or predictive efficiency. Within the PP framework, the system iteratively refines internal generative models to anticipate incoming related sensory inputs, thereby suppressing ascending prediction errors through precision-weighted inhibitory feedback. This is consistent with some empirical findings (e.g. Garrido et al. 2009; Ewbank et al. 2011), suggesting that when neuronal populations learn to predict low-level incoming signals from identical stimuli, they suppress related prediction errors. This corresponds to Bayesian model averaging that provides an optimal model that allows perceptual learning (FitzGerald et al. 2014); by decreasing the neural responses of repeated and expected inputs, the system preserves capacity for unexpected, relevant stimuli. The repetition suppression may be linked to self-modeling because information related to the self is more likely to be highly predicted and therefore suppressed. In fact, the repetition suppression paradigm has been used to distinguish self-representations and self-judgments from those related to others (Jenkins et al. 2008; Heleven and Van Overwalle 2019). For example, Heleven and Van Overwalle (2019) used a functional magnetic resonance imaging repetition suppression paradigm to identify neural regions that showed reduced activation when encoding repeated self-referential information.

## The predictive processing of the body-scan

### Non-action and refocusing as core attentional (precision) processes in meditation

The PP framework offers powerful means of bridging subjective experiences and computational processes underlying meditation techniques and experiences (see, for example, Deane et al. 2020; Laukkonen et al. 2023). While a number of these accounts discuss in passing the body-scan technique (see, in particular, Lutz et al. 2008; Lutz et al. 2019; Limanowski and Friston 2020; Laukkonen and Slagter 2021), the subtleties of this technique and the impact it can have on altering self-modeling are still largely underdeveloped. In what follows, we will aim to fill this gap in the research literature.

We will focus on two interdependent ways that a meditator places attention on an object, including the body: (i) they set the intention to maintain a still posture in order to sustain their attention on the chosen object (see Lutz et al. on non-action); and (ii) when their attention drifts from the intended object, they actively refocus attention away from the distraction and back to the object. These processes are maintained during the whole meditation session of the body-scan. Non-action is a matter of policy selection, of remaining in the sitting still position throughout the whole session (and subsequently the suspension of the usual policies of moving toward the desired and away from the undesired). This way, non-action is actually an active process—one actively selects and maintains the policy of non-action (Pagnoni and Guareschi 2021). As the influences of bottom-up prediction errors and top-down predictions are always in a conflicting relationship with regards to the inferential process, this act of attention also implies an active modulation of the precision or confidence of high-level priors on prediction error outcomes. For example, in the meditative context, the meditator actively engages in not moving when they feel certain urges, such as scratching their nose or changing their sitting position. The non-action process is facilitated by policies driving the cultivation of an open or non-judgmental attitude toward any arising experience during the session (e.g. thoughts, feelings, and sensations). In other words, non-action and the non-judgmental attitude can be seen as a voluntary unavailability to compulsive reactive patterns (Pagnoni and Guareschi 2021). This finds support in evidence suggesting that the body-scan can reduce cognitive and emotional reactivity (Cahn and Polich 2009; Cahn et al. 2010).

During meditation (and throughout the rest of our waking and dreaming life), multiple policies compete for attention selection at any given moment (cf. Cisek and Kalaska 2010; Clark 2013; Pezzulo et al. 2018). That means that the policy for non-action is inevitably overturned by other high-precision habitual (distracting) policies that represent the starting of mind-wandering. It follows that the practitioner must also improve their ability to monitor and refocus their attention when appropriate. As soon as the practitioner recognizes the mind-wandering process, they can learn to weaken the involuntary capture of attention by thoughts and experiences other than the chosen meditation object (Brewer et al. 2011; Laukkonen and Slagter 2021). As empirical studies on meditation show, refocusing attention activates brain regions linked to the voluntary control of thoughts and actions, including the premotor cortex and dorsal anterior cingulate cortex (for a review, see Fox et al. 2016).

### Focused attention and open monitoring in the body-scan

As mentioned in section “The body-scan meditation technique and its phenomenology,” the first phase of the body-scan consists of FA meditation on single body parts. In PP terms, FA has been described as setting high precision on the incoming sensory information concerning the target of the focus, leading to a decrease in sensory surprise (Lutz et al. 2019).

Here we apply this computational description of FA in the context of the body-scan. What is interesting about this first phase is that through FA, the practitioner gains increasingly accurate predictions about their body map and their internal states. Over time, the system is able to generate accurate low-level posteriors, resulting in an optimized body model (i.e. closer to sensory data) and more fine-grained interoceptive modeling. For example, this phase may allow the practitioner to disclose new information about previously unnoticed aspects and qualities of their body, such as a tilted posture or a sense of heaviness in part of the body. The reduction in uncertainty of the low-level predictions on somatosensory signals is supported by the PP model of endogenous attention presented in section “The body-scan meditation technique and its phenomenology” (e.g. Feldman and Friston 2010; Hohwy 2020). In this model, attention works as a kind of “searchlight” in the perceptual landscape, where the system places more weight on the prediction errors arising from the targeted sensory signals (Hohwy 2020:12). In this way, FA on the body enables somatosensory signals to carry more influence in perceptual inference, thereby allowing precision optimization of the somatosensory signals. That is why the repeated and consistent attention allocation on body parts during the first phase of the body-scan eventually leads to more accurate and available predictions about body maps and internal states. Neurophysiological studies seem to speak in favor of the sensory enhancing effect of attention, as attention is shown to increase cortical sensitivity (Reynolds et al. 2000; Pestilli and Carrasco 2005).

As one progresses in FA on the body, the more readily available body maps allow the practitioner to attend to larger bodily objects and eventually to the body as a whole. As the sensitivity to interoceptive and exteroceptive signals increases, the meditator may start recognizing larger patterns of dynamic bodily sensations occurring within or across their body areas. For example, if one feels a sense of heaviness somewhere in the body, after some time one may become aware that this feeling is constantly moving around the given area and perhaps happening at the same time as other sensations spread around the whole body.

This ability to better monitor experiences sets the stage for the second phase of the body-scan. In this phase, the practitioner is able to sense the whole structure of the body and related sensations easily and rapidly. We suggest that during this second phase, the practitioner senses their whole body *via* an OM mode of attention (for more detail, see section “The body-scan meditation technique and its phenomenology,” cf. Khin 1999; Vago and Silbersweig 2012). The non-preferential attention distribution typical of OM is applied to the dynamic landscape of sensations occurring throughout the body as a whole.

While FA assigns precision to the input relating to specific parts of the body (e.g. the head, the neck, etc.), OM meditation allows any and all data of one’s ongoing experience (parallel thoughts, sensations, etc.) to enter the scope of attention. This non-preferential way of distributing attention has been formulated as an overall reduction of relative gain on any ongoing inferences at different layers (prediction errors, priors, precision weighting, etc.) by Laukkonen and Slagter (2021). As the temporal span of high-level predictions decreases during the practice of OM, the overall counterfactual depth of one’s cognitive architecture decreases as well. In other words, by assigning relatively low precision to any source of experience, the system generates predictions less frequently and relies less on deep counterfactual models (for a discussion on affect-based attention in PP, see Ransom et al. 2020).

Building on this account, we suggest that the second phase of the body-scan serves to optimize the practitioner’s interoceptive model by flattening the priors’ landscape over sensations and body maps. Specifically, OM levels the salience of the prior distribution, decreasing the precision, frequency, and temporal span of high-level prior predictions on sensations and body maps (Laukkonen and Slagter 2021:205). In other words, the OM phase reduces the level of confidence over predictions (precision), the amount of predictions generated (the frequency) and the amount of time between one prediction and the next (the temporal span). As mentioned in section “Focused attention and open monitoring in the body-scan technique,” the practitioner is trained to regularly shift from OM to FA during the session (shifting to FA when losing free flow or meeting blind spots). This allows the system to reach a balance between boosting somatosensory processing through FA and reducing counterfactual depth of the body model through OM. In other words, as the meditator gets to know their single body parts better through FA, in the OM phase they may learn to “forget” or “let go” of prior expectations on the current internal state of their body.

The optimization of interoceptive modeling promoted by OM can be observed in pain research. Several studies show that OM practices can reduce the unpleasantness of pain without affecting its perceived intensity (Lutz et al. 2013; Zorn et al. 2020; Poletti et al. 2021). In the PP framework, pain is understood not merely as a direct reflection of bodily tissue damage but as an “overall estimate” of the threat given to the body from multiple sensory sources (Kiverstein et al. 2022:987). This means that pain serves as feedback informed by prediction error states, based on discrepancies between current sensory evidence and prior expectations about bodily integrity.

Taking a step further, we can say that pain intensity is tied to the system’s confidence in sensory prediction errors, while pain unpleasantness is linked to the system’s involuntary affective top-down evaluation on pain intensity (see Perlman et al. 2010 for a discussion), although whether the experience of pain has a constitutional affective component is a controversial issue (cf. Kim 1992; Chalmers 1997). In line with Laukkonen and Slagter (2021), we believe that OM practices likely reduce pain’s emotional impact by diminishing top-down evaluations while enhancing bottom-up somatosensory processing. OM modulates pain by shortening the temporal span of generative models, reducing the influence of high-level predictions that typically enhance pain unpleasantness, resulting in a less distressing pain experience (for a discussion, see Perlman et al. 2010).

As meditators of the body-scan gain expertise in the OM phase, they continue to sense pain but are less affected by the emotional reactions it usually triggers. This point is especially relevant when it comes to evaluating the therapeutic applications of the body-scan, particularly in reducing addiction-related behaviors and improving emotional regulation. Another way to put it is that as OM modulates the temporal depth of these generative models, the temporal depth itself becomes opaque (i.e. modeled) to the predictive system, making future and past expectations feel less real or self-relevant. As we will describe in the next section (specifically in section “Valence, addiction, and emotion regulation”), the opacification processes promoted by the body-scan may attenuate the dysfunctional feelings of craving or aversion linked to addictive behaviors, and broadly to maladaptive emotional regulation.

## Linking the body-scan to the sense of body boundaries dissolution

### Body attenuation through precision control

In this section, we extend the computational description that we provided of the body-scan to one of the most commonly reported experiences associated with the body-scan: the sense of body boundary dissolution, or bha${\dot{\textrm{n}}}$ga.

Up to this point, we have outlined how each attentional process involved in the body-scan technique can be described under the PP framework. In section “The predictive processing framework,” we explained that sensory attenuation occurs when predictions of a self-generated action match prediction errors of ongoing sensory data, resulting in a perceptually attenuated response (Auksztulewicz and Friston 2016; Grotheer and Kovács 2016). We described attention under PP as a mental action affecting hierarchical inference similarly to motor control (Metzinger 2004, 2017; Lutz et al. 2019; Limanowski and Friston 2020).

From this perspective, we can think of the attentional processes involved in the body-scan—e.g. non-action, refocusing attention, FA, and OM—as self-generated actions that produce expectancy effects for the “attentional movement” signals, predicting sensory consequences and reducing the salience of the sensations. In section “The predictive processing of the body-scan,” we argued that body-scan attentional processes (non-action, refocusing attention, FA, and OM) enhance the accuracy of low-level predictions. Briefly summarized, non-action and refocusing attention in the body-scan foster voluntary policies to accurately direct attention and sustain it for more extended periods. FA sharpens precision for targeted body parts, while OM optimizes the entire body’s somatosensory model, enhancing its ability to match sensory prediction errors. Therefore, the attentional strategies of the body-scan foster precision control over low-level predictions, which increasingly generate more accurate posteriors and consequently less discrepancy with ascendant prediction errors. This, we propose, facilitates a process of somatosensory adaptation and results in the subjective experience of feeling one’s body boundaries fading and eventually dissolving, or the state of bha${\dot{\textrm{n}}}$ga.

A series of studies on the interplay between attention and (somato) sensory attenuation seem to align with our hypothesis. While attention’s ability to enhance perception is well known (Treue and Trujillo 1999; Reynolds and Heeger 2009), its potential to attenuate sensory processing is often neglected (for a review, see Hughes et al. 2013. In particular, some adaptation effects have been observed in visual motion perception and in the “Troxler fading” illusion, in which an unchanging stimulus around a fixation point appears to fade over time (Lou 1999; Bonneh et al. 2014), especially in versions of the Troxler illusion where there is fading of the entire visual field, and not only at the periphery. As in the “Troxler fading” illusion, during the body-scan the arising sensations are targets of a prolonged attentional exposure, which can in turn promote sensory adaptation effects, i.e. the salience of the sensation gradually fades over time. Therefore, these factors may play a role in facilitating the PP enabled by the body-scan, which promote the sensory adaptation effects and the reported subjective experience of the body boundaries dissolution.

### Deep opacifying interoceptive modeling

So far, we have described how the interplay between attention and perception in the body-scan meditation can lead to the state of bha${\dot{\textrm{n}}}$ga. We now take a further step to consider how these interoceptive dynamics affect the modeling of the sense of self (i.e. the feeling of being oneself) and reports of so-called selfless experiences (i.e. an altered or diminished sense of self, for more detail, see section “Focused attention and open monitoring in the body-scan technique”). We are not taking a stand here about what the self is and how it can change in meditation, as it is a debatable issue that deserves its own space in a further manuscript (Miller, Becattini, *et al.*, in prep).

We propose that the deep interoceptive modeling promoted by the body-scan (as described in section “The predictive processing framework”) may also promote the “opacification” of processes involved in modeling different functions related to self-functioning at various scales. This may then lead to experiences commonly described by Buddhists as selfless states, and to key experiential insights sought after by practitioners, such as the realization of the impermanence of sensations (Anālayo 2012).

Experiential content that is transparently perceived is felt as immediately present and subjectively real (Metzinger 2017). However, when the co-construction of these experiences through perceptual mechanisms is accessed through meta-awareness, they can be said to become “opaque” to the experiencer. Examples of such opacification include lucid dreaming (being aware of dreaming), meta-awareness of thoughts (being aware of thinking, as opposed to being absorbed in mind-wandering), and pseudo-hallucinations (illusory sensations that are recognized as unreal) (Limanowski and Friston 2018). The opacification of the cognitive and phenomenological processes that shape experiential contents is a central skill developed through many forms of Buddhist (and non-Buddhist) meditation, particularly those that are said to foster “insight” into the nature of experience (Anālayo 2012).

In the specific case of the body-scan, practicing meditation modulates the automatic precision weightings on policies typically linked to embodied sensations. For example, an itch (embodied sensation) typically prompts scratching (automatic precision-weighted policy). However, by observing sensations without reacting (see non-action in section “Non-action and refocusing as core attentional (precision) processes in meditation”), these automatic precision assignments to action policies become increasingly opaque. Specifically, the body-scan as we have described it, this persistent “down-weighting of habitual and automatic trajectories of (pre) motor and autonomic reactions” (Lutz et al. 2019:2) increases learned control over two key processes: (i) habitual precision assignments and (ii) goal priors. This lays the ground for considering the potential positive effects of the body-scan for addiction and emotional regulation, as we will explain in the next subsection.

The first process, increasing control over habitual precision assignments, has an epistemic value. In PP, the epistemic value of a PP inference is related to the acquisition of new information that can enhance the model’s predictive capabilities over time (Lutz et al. 2008; Kaplan and Friston 2018; Tschantz et al. 2020). Through FA and OM, the body-scan allows the system to reduce the newsworthiness of bodily signals by increasing the sensitivity on somatosensory cues. As a result, the repeated reorientation of attention to the meditation target, or “refocusing of attention,” causes precision assignments to lose their sense of immediacy and feeling of realness (cf. Limanowski 2017; Metzinger 2017; Limanowski and Friston 2020). This learning of control over precision applies to both higher-level affective and cognitive dimensions, such as emotions or narratives, and lower-level ones, such as pain and itches. For instance, an experienced meditator may better recognize the fact that an involuntary emotion or narrative thought might automatically trigger a certain action policy, such as further thinking about an ex-partner.

The second process, increasing control over goal priors, has a pragmatic value. The pragmatic value of an action or action policy in PP is the likelihood that it will lead to a desired outcome or goal, e.g. maintaining a viable body temperature (Kaplan and Friston 2018). In the body-scan, non-action and refocusing attention policies train the practitioner to endogenously control the precision of goal priors, a training that eventually extends beyond the meditation session. Increased resilience to distractions reflects an improved ability to control precision on goal priors, refining attentional selection through mental action, similarly to refining motor commands through the iterative inference of control over action-outcome contingencies (Miall and Wolpert 1996; Wolpert and Flanagan 2001). For example, an experienced meditator may better respond to distressing emotions outside the meditation session because of a learned tracking of the policy selection.

Ultimately, the learned control of precision on bodily signals *via* opacification may lead to insights about the impermanent and co-constructed qualities of the body and self-models. The sense of self is a multidimensional experience including a spectrum of processes ranging from more minimal, momentary, and embodied to more narrative, temporally extended, and abstract dimensions (Gallagher et al. 2024). The embodied sense of self, in particular, is structured by moment-to-moment fluctuations in sensory events (for a more detailed discussion, please see Colombetti 2014; Kiverstein et al. 2019). Observing these precision allocations independently of agentive engagement disrupts the usual transparency of self-inference, revealing the contingent nature of feeling oneself. This kind of experience is usually described by Buddhists as states with a decreased sense of self and an increase in experiential insights, such as the realization of the impermanence of sensations (Anālayo 2012).

### Valence, addiction, and emotion regulation

Here we propose to examine potential positive effects of the body-scan for addiction dynamics. Just as they attenuate the sense of body boundaries, the opacification processes promoted by focusing on bodily signals may attenuate the typical feelings of craving or aversion linked to addictive behaviors.

The way we act and interact in the world depends on the evaluation of the affective significance of predicted sensory signals, i.e. on our valence system (see also Rietveld 2008). Addiction dynamics are tightly connected with the valence and reward system (Ahmed 2004). We have recently linked reward value in predictive error minimization (PEM) with positive affective valence (Kiverstein et al. 2019; see also Joffily and Coricelli 2013; Van de Cruys 2017). But what does it mean for something to possess affective value? In the context of PEM, drives are defined by the discrepancy between expected and actual states. Reward value pertains to the organism’s drive to diminish some form of uncertainty, particularly concerning homeostatic bounds (den Ouden et al. 2010; FitzGerald et al. 2014). For instance, money holds reward value because it allows us to be certain that our bills are paid monthly, thereby reducing uncertainty. Positive and negative valence arise when errors are reduced more or less efficiently than anticipated (Miller et al. 2022; cf. Polani 2009). This does not imply that less error necessarily feels better. For example, if a reduction in error is highly expected, such as the relief from scratching an itch, little to no positive valence would emerge.

The main point of this account is that we do not only track error production and reduction but also the rate at which we have historically been able to reduce error in given circumstances. This means we have an intrinsic drive to not only reduce error but to do so at an increasingly efficient rate (Joffily and Coricelli 2013). In other words, we strive for optimization. This perspective advances our understanding of reward by specifying precisely what the reward system is monitoring and orienting its processing toward.

Under this PEM framework, drug addiction can be described as hijacking the system’s error sensitivity (Keramati and Gutkin 2013), leading the system to self-organize in relation to the environment in ways that run counter to continued thriving. Sensitivity to the rise and fall in error reduction plays a crucial role in helping an agent to balance the multiple relevant affordances, staying in touch with many of the possibilities that matter to them when something in the agent’s situation changes (Miller et al. 2020). In certain contexts, some substances can progressively drive behavior to the neglect of other possibilities that are also of concern to the agent. They do so because they make it seem to the agent as if an improvement has taken place in how well the organism is improving its predictive grip.

Some drugs produce strong and immediate feelings of pleasure through an influx of dopamine. Each time they are consumed, the increase in pleasure signals to the organism that something better than expected has taken place. Unfortunately, the temporary state of chemically scaffolded grip wears off all too quickly, only to be replaced with more error and uncertainty. Meanwhile, the drug user may be progressively losing touch with the other things they care about. The production of dopamine caused by the drug makes it seem to the organism as if the policy of seeking and using the drug is the most reliable way of getting itself into the states it expects to occupy. Thus, the policy of seeking and using the substances soon comes to be the policy the organism has the most confidence in. Schwartenbeck et al. (2015) hypothesize that the generative model in repeated drug users owes its suboptimality to the precision that is assigned to the users’ habitual behaviors. Over time, as these ways of self-organizing are increasingly over-learned, the unthinking—interoceptive-driven—behavioral dispositions are enacted more and more consistently, produced by a greater and greater number of drug-related cues, while other opportunities for rewarding behavior are increasingly muted. In this context, we want to propose the case of drug addiction as a field where deep interoceptive modeling can have a positive impact.

We suggest that addiction dynamics can be powerfully impacted by the body-scan practice (cf. Miller et al. 2020; for reviews, see Zgierska et al. 2009; Kadri et al. 2020). In addiciton, habitual behaviors become more highly expected, which in turns make goal-directed behaviors become less highly expected. Drugs that induce strong reward signals facilitate this imbalance by re-tuning the confidence that some policy of behaviors will result in expected sensory states. This re-tuning happens through a sensitivity to error dynamics which can potentially be regulated by the body-scan technique.

In particular, we propose that practicing the body-scan may help reduce the urgency (valence) associated with addictive behaviors by increasing sensitivity to prediction errors related to bodily sensations. As the body-scan models these sensations, it may lessen the power of cravings by rendering them more available to awareness. Over time, this process can “opacify” the valence system, diminishing its ability to evoke a strong sense of urgency (in Buddhist parlance, craving or taṇhā) linked with self-identification (Metzinger 2004). Instead of fulfilling the prediction, the system learns to accept prediction errors, making them reducible through perceptual rather than active inference. This process involves controlling the precision of goal priors and bodily signals, allowing the system to disentangle the influence of sensations habitually driving craving or aversion.

As a last point, we propose to link the body-scan’s opacifying processes to emotion regulation. Good interoceptive abilities are usually connected with adaptive strategies of emotion regulation (Füstös et al. 2013; Mallorquí-Bagué et al. 2016; for a review, see Brewer et al. 2011), lower psychological distress (Bohlmeijer et al. 2010), anxiety, depression (Hofmann et al. 2010), and somatic symptoms (Fjorback et al. 2013). Although there is mixed evidence on the issue, and enhanced interoception has led in some cases to panic (see Yoris et al. 2015), disrupted interoceptive accuracy is often associated with emotion regulation difficulties (Paulus and Stein 2010; Dijkstra et al. 2024; for a discussion, see Britton et al. 2021). In particular, low interoceptive accuracy has been linked to increased anxiety and alexithymia (difficulties in recognizing, expressing, and describing one’s emotions) (for a review, see Van Bael et al. 2024), due to the “noisiness” of internal data—poor access to internal states confused with other signals, making interoception noisy. Uncertainty in noisy environments likely increases stress, negatively impacting physiological processes and leading to emotional disturbance (Nord and Garfinkel 2022:506). Since the brain predicts both external and internal sensory signals, interoceptive inferences are crucial for emotional experiences, psychopathology symptoms, and self-awareness (see also Seth et al. 2012; Limanowski and Blankenburg 2013; Ciaunica et al. 2022).

There is a growing body of literature exploring the complex link between mindfulness and interoceptive processes (Hölzel et al. 2011; Mirams et al. 2013; Garfinkel et al. 2015; Treves et al. 2019; Lima-Araujo et al. 2022; Nath et al. 2025). For example, while Vipassana meditators do not exhibit greater interoceptive accuracy (sensory detection ability) compared to controls, they show enhanced interoceptive awareness (meta-cognitive awareness of accuracy) (Khalsa et al. 2008), somatic sensitivity (Fox et al. 2012), and show structural changes in the insula, a brain region involved in interoceptive tasks (see Farb et al. 2013, Lippelt et al. 2014, and Ganesan et al. 2022 for reviews).

If meditating on the body can facilitate interoceptive awareness, then this practice may provide a precise and controllable internal experience, reducing interoceptive noise, and thereby fostering emotion regulation. This is in line with our argument insofar as the body-scan does not lead the meditator to constantly feel an overly sensitive body after the meditation session, a condition that would be closer to psychopathology (Brown et al. 2013). Rather, what we have argued is that the increase in accuracy on body priors (i.e. an overly sensitive body) due to the body-scan occurs during the meditation session only, as a condition possibly contributing to the dissolution of bodily boundaries. In the present section, instead, we have attempted to outline potential positive effects of the body-scan after the meditation session, i.e. when one is not actively meditating, by pointing to the role of opacification processes for modulating interoceptive pathways implicated in regulating addictive behaviors.

It is also important to highlight the risks connected with the potential therapeutic use of meditation (cf. Cebolla et al. 2017; Lindahl et al. 2017). For example, Britton (2019) points out that focusing on negative emotions and persistently turning toward difficult stimuli may have negative consequences. Meditation can sometimes lead to distressing and destabilizing experiences of depersonalization and dissociation, especially when practiced intensively without adequate supervision (Cebolla et al. 2017; Lindahl et al. 2017; Britton et al. 2021; Deane et al. 2020; Ciaunica et al. 2022).

## Conclusions

This article aims to provide a mechanistic understanding of the body-scan meditation technique through the lens of the PP framework. We integrated key insights from perceptual and attentional models in PP theory (Friston 2005; Adams et al. 2013; Clark 2013; Hohwy 2013) with existing theoretical accounts of meditation (Lutz et al. 2019; Deane et al. 2020; Laukkonen and Slagter 2021; Pagnoni and Guareschi 2021), phenomenological studies (Berkovich-Ohana et al. 2013; Ataria et al. 2015; Nave et al. 2021), and empirical findings (Hughes et al. 2013; Garfinkel et al. 2015; Dambrun 2016; Treves et al. 2019; Lima-Araujo et al. 2022).

We proposed that the body-scan relies on four interdependent attentional processes—non-action, refocusing attention, FA, and OM—which collectively enhance the accuracy of the lower-level predictions related to somatosensory cues. The non-action and refocusing attention policies, maintained in parallel to the other two processes, reduce the precision over sensations and thoughts that drive the selection of other (distracting) policies. In the first phase of the body-scan, the meditator practices FA on single parts of the body, which increases the confidence on low-level prediction error states and the gain on bottom-up processes. In the second phase of the body-scan, when the meditator attends to their body as a whole and practices OM, the precision, frequency, and temporal span of high-level prior predictions decrease. These precision control processes, we argue, lead the system to generate less discrepancy between the body model (posteriors) and the incoming signals, a mechanism often referred to as sensory adaptation, in which sensory signals from the body become less prominent over time, culminating in a decreased perception of body boundaries. This mechanism aligns with reports from practitioners describing the gradual dissolution of body boundaries, often referred to as the state of bha${\dot{\textrm{n}}}$ga. We further explored how this process may contribute to key experiential insights in meditation. Our model suggests that the cultivation of interoceptive awareness may “opacify” the processes of perception that come to shape one’s experience of the world, and in this way liberate the practitioner from involuntary valenced drives that reinforce a rigid sense of self.

Finally, we highlighted the potential of the body-scan for therapeutic applications, particularly in reducing addiction-related behaviors and improving emotional regulation. By increasing precision over prediction errors related to bodily sensations, the body-scan may weaken the urgency of cravings and reduce emotional reactivity. However, we also emphasized the importance of caution in applying meditation techniques in mental health interventions, as meditation can have unintended effects if not used carefully, particularly in individuals with a history of trauma or psychological distress.

While our account offers a neurocomputational explanation for the body-scan’s effects, we acknowledge that other factors may also play a role. In particular, holding a culturally sanctioned expectation that meditation will lead to particular experiences, such as the dissolution of bodily boundaries, may generate these kinds of experiences through top-down effects on perception (Lifshitz et al. 2013; Cassaniti and Luhrmann 2014; Lifshitz 2018). Future research is needed to explore these factors and refine our understanding of how attentional processes in meditation interact with broader sociocultural contexts.

In conclusion, the body-scan represents a powerful technique for training attention and interoceptive awareness. By leveraging PP principles, we have outlined a mechanistic explanation for how this practice modulates the experience of the bodily self, offering valuable insights for both contemplative science and potential therapeutic applications.

## Data Availability

There are no new data associated with this article.
